# Association of geographical disparities and segregation in regional treatment facilities for Black patients with aneurysmal subarachnoid hemorrhage in the United States

**DOI:** 10.3389/fpubh.2024.1341212

**Published:** 2024-05-10

**Authors:** Jean-Luc K. Kabangu, Lane Fry, Adip G. Bhargav, Frank A. De Stefano, Momodou G. Bah, Amanda Hernandez, Adam G. Rouse, Jeremy Peterson, Koji Ebersole, Paul J. Camarata, Sonia V. Eden

**Affiliations:** ^1^Department of Neurological Surgery, University of Kansas Medical Center, Kansas City, KS, United States; ^2^University of Kansas School of Medicine, Kansas City, KS, United States; ^3^Michigan State University College of Human Medicine, East Lansing, MI, United States; ^4^University of Michigan Medical School, Ann Arbor, MI, United States; ^5^Department of Neurosurgery, Semmes Murphey Clinic, Memphis, TN, United States; ^6^Department of Neurological Surgery, University of Tennessee Health Science Center, Memphis, TN, United States

**Keywords:** regional disparities, Black patients, aneurysmal subarachnoid hemorrhage, segregation, healthcare disparities, equitable care

## Abstract

**Background and objectives:**

This study investigates geographic disparities in aneurysmal subarachnoid hemorrhage (aSAH) care for Black patients and aims to explore the association with segregation in treatment facilities. Understanding these dynamics can guide efforts to improve healthcare outcomes for marginalized populations.

**Methods:**

This cohort study evaluated regional differences in segregation for Black patients with aSAH and the association with geographic variations in disparities from 2016 to 2020. The National Inpatient Sample (NIS) database was queried for admission data on aSAH. Black patients were compared to White patients. Segregation in treatment facilities was calculated using the dissimilarity (D) index. Using multivariable logistic regression models, the regional disparities in aSAH treatment, functional outcomes, mortality, and end-of-life care between Black and White patients and the association of geographical segregation in treatment facilities was assessed.

**Results:**

142,285 Black and White patients were diagnosed with aSAH from 2016 to 2020. The Pacific division (*D* index = 0.55) had the greatest degree of segregation in treatment facilities, while the South Atlantic (*D* index = 0.39) had the lowest. Compared to lower segregation, regions with higher levels of segregation (global *F* test *p* < 0.001) were associated a lower likelihood of mortality (OR 0.91, 95% CI 0.82–1.00, *p* = 0.044 vs. OR 0.75, 95% CI 0.68–0.83, *p* < 0. 001) (*p =* 0.049), greater likelihood of tracheostomy tube placement (OR 1.45, 95% CI 1.22–1.73, *p* < 0.001 vs. OR 1.87, 95% CI 1.59–2.21, *p* < 0.001) (*p* < 0. 001), and lower likelihood of receiving palliative care (OR 0.88, 95% CI 0.76–0.93, *p* < 0.001 vs. OR 0.67, 95% CI 0.59–0.77, *p* < 0.001) (*p* = 0.029).

**Conclusion:**

This study demonstrates regional differences in disparities for Black patients with aSAH, particularly in end-of-life care, with varying levels of segregation in regional treatment facilities playing an associated role. The findings underscore the need for targeted interventions and policy changes to address systemic healthcare inequities, reduce segregation, and ensure equitable access to high-quality care for all patients.

## Introduction

Black patients in America face significant challenges within the healthcare system, resulting in disparities that manifest as higher rates of chronic diseases, limited access to quality healthcare, and worse health outcomes compared to their White counterpart (1, 2). These disparities are evidenced by elevated rates of chronic diseases, restricted access to high-quality healthcare, and poorer health outcomes among Black patients compared to their White counterparts (2–8). Moreover, these disparities extend beyond general health conditions to acute medical crises, notably in neurosurgical emergencies like aneurysmal subarachnoid hemorrhage (aSAH). For instance, studies have indicated that Black patients with aSAH experience higher mortality rates and greater disability compared to White patients (9–11).

The root causes of these health disparities are multifaceted. They include limited access to healthcare, socioeconomic inequalities, cultural barriers, implicit biases, and disparities in the quality of care (3, 12–14). These factors are further compounded by geographical variations, with differences in healthcare infrastructure, socioeconomic conditions, cultural norms, healthcare provider demographics, and historical patterns of segregation (5, 6, 15). Segregation in healthcare refers to the unequal distribution of medical services, resources, and opportunities based on factors such as race, ethnicity, socioeconomic status, or geographic location. It can lead to significant disparities in access to quality medical services and resources, resulting in marginalized communities receiving substandard care and experiencing worse health outcomes. Hobar et al. explicated segregation in regional neonatal intensive care units (NICU) among Black and White newborns, with Black babies being treated at lower quality NICUs than White neonates (3, 16).

The legacy of segregation in healthcare for Black Americans is marked by a history of systemic discrimination and exclusion. In the Jim Crow era, legally sanctioned racial segregation relegated Black patients to separate, “colored” facilities, which were typically plagued by chronic underfunding and a dearth of resources, resulting in care that was markedly inferior to that available in institutions serving White patients. These “Negro hospitals,” as they were then known, became symbols of the broader injustices of the time—enduring emblems of inequality in American healthcare (17–22). Although the overt legal structures of segregation have been dismantled, the shadows of these historical disparities continue to loom over contemporary healthcare outcomes for Black patients (19–22).

It is within this context that our study examines the relationship between the continued segregation in healthcare settings and its impact on the treatment and outcomes of Black patients suffering from aSAH. Existing research indicates that minority groups with aSAH, including Black patients, often receive more aggressive medical interventions, like tracheostomy and gastrostomy tube placements, as well as blood transfusions, than their White counterparts (23). In contrast, these patients are less frequently involved in palliative care consultations or designated with Do Not Resuscitate (DNR) status (23). We hypothesize a direct correlation between the degree of segregation in treatment facilities and the observed disparities in care. Specifically, we focus on the prevalence of aggressive treatment interventions and the lack of engagement with palliative care services and DNR status in areas with higher segregation indices. By shedding light on the pervasive nature of these disparities and their association with segregation, this study seeks to articulate the ongoing challenges in achieving healthcare equity. Furthermore, it aspires to lay the groundwork for interventions specifically designed to counteract these disparities and foster a more equitable healthcare landscape for Black patients with aSAH.

## Methods

### Data source

The National Inpatient Sample (NIS) database was queried from 2016 to 2020 for diagnosis of ruptured aSAH using the International Classification for Disease version 10 (ICD-10). Patients with ICD - 10 codes 160.00–160.09 met the primary inclusion criteria (Supplementary Table S1). Patients with traumatic SAH (*n =* 1,740) or SAH associated with an arteriovenous malformation (*n =* 23,575) were excluded from the study (Supplementary Figure S1). This approach aligns with the study’s aim to investigate healthcare disparities and outcomes in aSAH, distinct in its pathophysiology and treatment from conditions like traumatic SAH and SAH due to arteriovenous malformations. By concentrating on aSAH, our study aligns with existing literature and avoids the confounding variables introduced by the inclusion of other SAH etiologies, thereby ensuring a more accurate and homogenous examination of the specific disparities and outcomes in this patient group. The NIS, part of the Healthcare Cost and Utilization Project (HCUP), is a vast database containing de-identified inpatient hospitalization data from a wide array of U.S. hospitals, enhancing its national representativeness. It utilizes discharge weights from participating hospitals to provide nationally representative estimates, facilitating diverse healthcare research. However, limitations include potential billing and coding inaccuracies, variations in hospital reporting practices, and the exclusion of certain facilities like federal hospitals. Additionally, its focus is solely on inpatient data, excluding outpatient care. Due to the de-identified, retrospective nature of this study, Institutional Review Board approval was not sought.

### Population

The NIS database stratifies race into 6 groups: White, Black, Hispanic, Asian/Pacific Islander, Native American, and Other. Only patients listed as Black or White were included in this study. Our analysis focuses on Black and White patients to closely examine the significant disparities predominantly observed between these racial groups. This targeted approach not only captures a vital element of the wider discourse on racial inequalities in healthcare but also acknowledges the enduring impact of historical segregation and systemic biases. These long-standing issues have disproportionately affected Black communities in the United States, perpetuating a cycle of healthcare disparities. Patients were further divided by geographical location using NIS-provided United States Census divisions (New England, Middle Atlantic, East North Central, West North Central, South Atlantic, East South Central, West South Central, Mountain, and Pacific). Supplementary Table S2 outlines the corresponding states that make up each U.S. Census division.

### Outcomes and covariates

Research questions centered on treatment disparities, functional outcomes, mortality, and end-of-life care. Treatment was dichotomized into two groups for patients undergoing aneurysm treatment (via open surgical clipping or endovascular therapy) and patients who did not receive either treatment. Functional outcomes in SAH cases were assessed using the NIS-SAH Outcomes Measure (NIS-SOM), a validated instrument provided by the NIS data source. This comprehensive metric evaluates treatment effectiveness and forecasts overall patient prognosis by integrating clinical, demographic, and hospital-related variables, similar to the approach used in modified Rankin scores. The NIS-SOM, is a dichotomous tool that classifies patient outcomes post-discharge into two categories: “good outcome” and “poor outcome.” A “good outcome” signifies a patient’s discharge to their home or a rehabilitation facility, reflecting a positive recovery path. In contrast, a “poor outcome” includes a spectrum of less favorable scenarios, such as in-hospital mortality, or discharge to a facility offering nursing care, extended care, long-term acute care, or hospice services. The design of the NIS-SOM aims to efficiently delineate patient recovery levels and care needs at the point of discharge, offering a clear and structured measure for evaluating SAH patient outcomes (24). To investigate disparities in end-of-life care, we examined the racial differences in life-sustaining interventions (mechanical ventilation, tracheostomy tube placement, gastrostomy tube placement, and blood transfusions), the utilization of palliative care services, and DNR. All analyses included the following covariates: age, sex (male and female), admission year, hospital size, teaching status, primary expected payer (Medicare, Medicaid, private insurance, self-pay, no charge, other), hypertension, obesity, smoking status, coronary artery disease, chronic kidney disease, hyperlipidemia, diabetes mellitus, alcohol abuse, atrial fibrillation, and the NIS SAH Severity Score (NIS-SSS) (25, 26). The NIS-SSS is a scoring system derived from the NIS database to assess the severity of aSAH using clinical and demographic data. Validated against established grading systems like the Hunt and Hess scale, it provides a reliable tool for stratifying patients and analyzing outcomes in aSAH studies (27). Outcomes and covariates not directly provided by the NIS were derived using secondary discharge diagnoses in the data source (Supplementary Table S2).

### Measure of segregation

**Figure 1:**
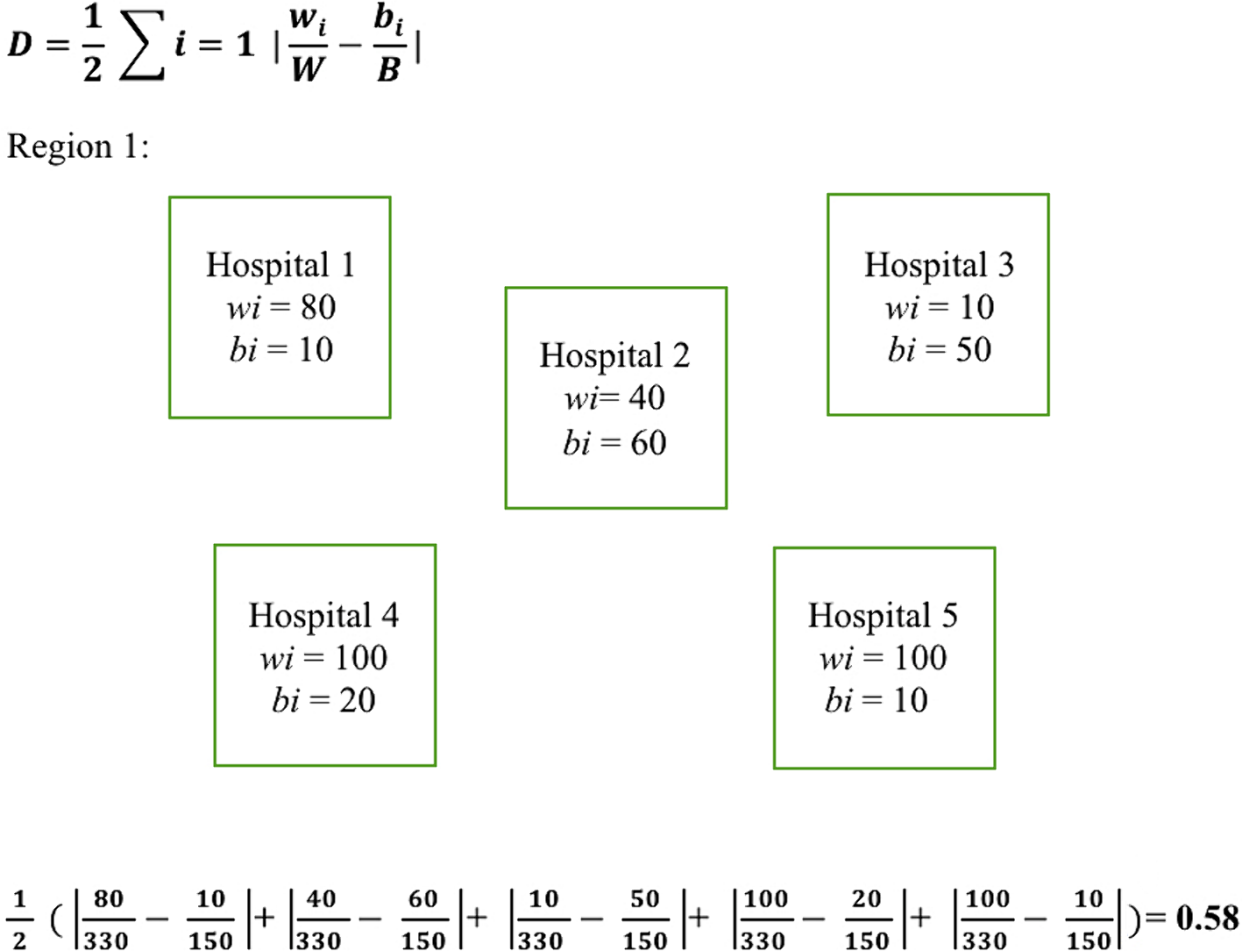
Dissimilarity index equation with example calculation. Illustrates the equation used for calculating the *D* index along with a sample calculation. Here, *w*_i_ and *b_i_* represent the number of White and Black patients treated at a given hospital within a region, respectively. *W* and *B* denote the total number of White and Black patients respectively within the region that the *D* index is being calculated.

To measure segregation in treatment facilities between Black and White patients within each U.S. Census region, we calculated the Duncan dissimilarity (D) index as outlined by Austin et al. (7). The *D* index is a statistical measure used to quantify the degree of segregation between two groups within a geographic area. It measures the proportion of individuals from one group who would need to change their location to achieve an even distribution with the other group. The *D* index ranges from zero to one, where zero indicates complete integration or no segregation, and one represents complete segregation. The *D* index was chosen for this study due to its established reliability, as demonstrated by its application by the U.S. Census Bureau for assessing residential segregation, and its successful use in previous research to quantify racial segregation in healthcare facilities, making it an ideal measure for exploring segregation’s impact on healthcare disparities (7, 28–30). The formula used for calculating the *D* index along with an example calculation is illustrated in Figure 1. *D* indexes were calculated by U.S. census region for each of the studied years.

### Statistical analysis

Univariate analysis was performed to explore differences in rates of mechanical ventilation, tracheostomy tube placement, gastrostomy tube placement, blood transfusions, the utilization of palliative care services, and DNR status for race (Black vs. White) and region (Supplementary Table S4). For variables with *p* < 0.20, multivariable logistic regression models were performed to control for covariate influence. All multivariable models used the above-outlined covariates. Patients missing data on variables of interest were excluded from the analyses. Racial disparities were compared to the national average for each of the nine census divisions. Analysis of variance (ANOVA) was used to compare differences in the level of segregation as measured by the *D* index among the regions. Finally, the impact of the level of segregation on the outcomes of interest was tested by comparing high (*D* index of 0.50 or higher) and low *D* index (*D* index <0.50) regions using multivariable logistic regression with the included covariates followed by a global *F* test when both regions showed statistical significance. If the overall *F* test was statistically significant, then high and low *D* index regions were compared using a *t*-test for the outcome of interest. All tests for significance were two-sided, with a *p-*value of 0.05 or less defined as statistically significant. This threshold of 0.05 was chosen to align with the convention used in other publications utilizing the NIS Database, ensuring consistency in our approach and facilitating comparisons with similar studies (23). Statistical analysis was performed using R version 4.2.2. This study adheres to The Strengthening the Reporting of Observational Studies in Epidemiology (STROBE) initiative recommendations (31).

## Results

We included 142,285 (113,060 White, 29,255 Black) patients hospitalized with aSAH from 2016 to 2020 in this study. Most Black patients in the cohort were cared for in the South Atlantic (33%), East North Central (16%), and Middle Atlantic (15.90%) regions. Similar observations were noted with White patients: South Atlantic (20%), East North Central (18%), and Middle Atlantic (13%). Table 1 provides a summary of baseline characteristics for the study cohort.

**Table 1:** Patient and hospital demographics by race.

Characteristic		White		Black	
		**No.**	**(%)**	**No.**	**(%)**
Total number of patients		113,060	79	29,225	21
Sex					
	Male	50,410	45	11,915	41
	Female	62,650	55	17,310	59
Region					
	New England	6,315	6	690	2
	Middle Atlantic	14,570	13	3,970	14
	East North Central	19,835	18	4,665	16
	West North Central	7,860	7	980	3
	South Atlantic	22,400	20	9,765	33
	East South Central	9,375	8	2,835	10
	West South Central	10,365	9	3,420	12
	Mountain	8,025	7	600	2
	Pacific	14,315	13	2,300	8
Comorbidity					
	Chronic kidney disease	12,595	11	5,410	19
	Atrial fibrillation	185	0.16	15	0.05
	Coronary artery disease	18,290	16	2,985	10
	Alcohol abuse	2,550	2	790	3
	Hypertension	60,670	54	14,860	51
	Diabetes mellitus	22,495	20	7,645	26
	Obesity	12,885	11	4,510	15
	Hyperlipidemia	39,280	35	7,975	27
	Congestive heart failure	14,375	13	4,535	16
	Smoking	23,145	20	4,555	16
	Admission GCS < 8	4,635	4	1,280	4
Bed size					
	Small	10,550	9	2,565	9
	Medium	24,925	22	6,795	23
	Large	77,585	69	19,865	68
Primary expected payer					
	Medicare	60,395	53	11,555	40
	Medicaid	11,530	10	6,670	23
	Private insurance	33,380	30	7,860	27
	Self-pay	4,245	4	2,080	7
	No charge	230	0.20	175	0.60
	Other	3,280	3	885	3

### Regional disparities in treatment, functional outcomes, and mortality

Nationally, Black patients had higher treatment rates following aSAH in comparison to White patients (OR 1.10, 95% CI 1.02–1.18, *p* = 0.013). However, significant racial differences in undergoing aSAH treatment were only demonstrated in the East South-Central region (OR 1.52, 95% CI 1.24–1.88, *p* < 0. 001). Black patients had worse NIS-SOM outcomes on the national level than White patients (OR 1.10, 95% 1.02–1.19, *p* = 0.010), with significantly worse functional outcomes seen in the Middle Atlantic (OR 1.27, 95% CI 1.04–1.55, *p* = 0.022) and West South-Central regions (OR 1.27, 95% CI 1.03–1.56, *p* = 0.025). Nationally, the mortality rate for Black patients was lower than that of White patients following aSAH (OR 0.81, 95% CI 0.75–0.88, *p* < 0.001). Black patients in the East North Central (OR 0.68, 95% CI 0.55–0.83, *p* < 0.001), East South Central (OR 0.63, 95% CI 0.47–0.84, *p* = 0.002), and West South Central (OR 0.74, 95% CI 0.58–0.96, *p* = 0.022) demonstrated statistically significant lower likelihood of mortality.

### Geographical variance in disparities in end-of-life care

Black patients were twice as likely as White patients to have tracheostomy tube placement in the Pacific division (OR 2.17, 95% CI 1.46–3.22, *p* < 0.001). Black patients in the Middle Atlantic (OR 1.76, 95% CI 1.27–2.43, *p* < 0.001), East North Central (OR 1.83, 95% CI 1.39–2.42, *p* < 0.001), and West South Central (OR 1.83, 95% CI 1.39–2.42, *p* < 0.001) regions had higher likelihood of tracheostomy tube placement in comparison to the national level (OR 1.67, 95% CI 1.48–1.89 *p* < 0.001). Conversely, a lower likelihood of tracheostomy placement than the national average was seen in the South Atlantic (OR 1.33, 95% CI 1.05–1.68, *p* = 0.018) and East South Central (OR 1.61, 95% CI 1.10–2.35, *p* = 0.015) regions. Black patients in the New England region had the greatest likelihood of gastrostomy tube placement (OR 2.00, 95% CI 1.13–3.33, *p* = 0.017), while having the least likelihood of receiving palliative care services (OR 0.42, 95% CI 0.22–0.80, *p* = 0.008) and utilization of DNR (OR 0.47, 95% CI 0.27–0.85, *p* = 0.012). Black patients in the South Atlantic had the lowest degree of disparity in the likelihood of gastrostomy tube placement (OR 1.36, 95% CI 1.14–1.61, *p* = 0.001) and receipt of palliative care (OR 0.76, 95% CI 0.65–0.89, *p* = 0.001). Table 2 summarizes the disparities for Black patients compared to White patients by region in treatment, outcomes, mortality, and end-of-life care.

**Table 2:** Odds ratios for treatment, functional outcomes, and end-of-life care for Black patients by region.

	Treatment			NIS-SOM		
Region	OR	95% CI	*p-*value	OR	95% CI	*p-*value
New England	0.74	0.43–1.27	0.272	1.50	0.96–2.34	0.075
Middle Atlantic	1.16	0.94–1.43	0.177	1.27	1.04–1.55	**0.022**
East North Central	0.89	0.75–1.06	0.180	1.16	0.94–1.43	0.178
West North Central	1.04	0.70–1.54	0.842	0.89	0.54–1.48	0.663
South Atlantic	1.05	0.91–1.20	0.504	1.01	0.88–1.15	0.903
East South Central	1.52	1.24–1.88	**<0.001**	1.14	0.88–1.47	0.316
West South Central	1.17	0.94–1.45	0.156	1.27	1.03–1.56	**0.025**
Mountain	0.67	0.40–1.12	0.124	0.89	0.60–1.31	0.543
Pacific	1.15	0.91–1.44	0.235	1.04	0.83–1.31	0.725
National	1.10	1.02–1.18	**0.013**	1.10	1.02–1.19	**0.010**

	**Mortality**			**Mechanical ventilation**		
Region	OR	95% CI	*p-*value	OR	95% CI	*p-v*alue
New England	1.28	0.81–2.03	0.288	9825.19	28.35–3405687.98	**0.002**
Middle Atlantic	0.89	0.72–1.10	0.277	1.20	0.49–2.94	0.686
East North Central	0.68	0.55–0.83	**< 0.001**	1.85	1.07–3.21	**0.028**
West North Central	1.06	0.66–1.68	0.813	0.06	0.01–0.50	**0.010**
South Atlantic	0.98	0.85–1.13	0.781	1.52	0.64–3.59	0.34
East South Central	0.63	0.47–0.84	**0.002**	1.7	0.83–3.49	0.149
West South Central	0.74	0.58–0.96	**0.022**	0.96	0.30–3.05	0.943
Mountain	0.67	0.36–1.22	0.188	1.7	0.51–5.67	0.39
Pacific	0.79	0.61–1.01	0.059	0.92	0.33–2.56	0.868
National	0.81	0.75–0.88	**<0.001**	1.3	0.89–1.89	0.172

	**Tracheostomy**			**Gastrostomy**		
Region	OR	95% CI	*p-*value	OR	95% CI	*p-*value
New England	1.84	0.86–3.93	0.115	2.00	1.13–3.53	**0.017**
Middle Atlantic	1.76	1.27–2.43	**0.001**	1.89	1.43–2.51	**< 0.001**
East North Central	1.83	1.39–2.42	**< 0.001**	1.66	1.31–2.11	**<0.001**
West North Central	1.38	0.72–2.66	0.328	1.50	0.89–2.54	0.128
South Atlantic	1.33	1.05–1.68	**0.018**	1.36	1.14–1.61	**0.001**
East South Central	1.61	1.10. - 2.35	**0.015**	1.70	0.83–3.49	0.149
West South Central	1.86	1.29–2.66	**0.001**	1.95	1.44–2.63	**< 0.001**
Mountain	1.95	0.93–4.06	0.075	0.98	0.49–1.96	0.945
Pacific	2.17	1.46–3.22	**< 0.001**	1.52	1.11–2.07	**0.009**
National	1.67	1.48–1.89	**<0.001**	1.58	1.43–1.74	**<0.001**

	**Transfusions**			**Palliative care**		
Region	OR	95% CI	*p-*value	OR	95% CI	*p-*value
New England	0.69	0.27–1.74	0.428	0.42	0.22–0.80	**0.008**
Middle Atlantic	1.61	1.19–2.20	**<0.001**	0.78	0.61–1.01	0.059
East North Central	1.37	0.96–1.93	0.079	0.67	0.53–0.84	**0.001**
West North Central	2.38	0.95–5.95	0.064	0.76	0.46–1.23	0.261
South Atlantic	1.23	0.98–1.55	0.072	0.76	0.65–0.89	**0.001**
East South Central	1.74	1.06–2.86	**0.029**	0.59	0.43–0.80	**0.001**
West South Central	1.79	1.18–2.70	**0.006**	0.67	0.51–0.87	**0.003**
Mountain	1.81	0.83–3.95	0.135	0.57	0.32–1.02	0.059
Pacific	1.25	0.82–1.90	0.304	0.68	0.51–0.91	**0.008**
National	1.49	1.30–1.70	**<0.001**	0.69	0.63–0.75	**<0.001**

	**Do not resuscitate**					
Region	OR	95% CI	*p-value*			
New England	0.47	0.27–0.85	**0.012**			
Middle Atlantic	0.75	0.59–0.96	**0.02**			
East North Central	0.60	0.49–0.96	**0.001**			
West North Central	0.71	0.46–1.09	0.116			
South Atlantic	0.70	0.60–0.82	**<0.001**			
East South Central	0.55	0.42–0.73	**<0.001**			
West South Central	0.71	0.55–0.91	**0.008**			
Mountain	0.52	0.30–0.91	**0.019**			
Pacific	0.75	0.57–0.99	**0.044**			
National	0.66	0.61–0.72	**<0.001**			

NIS-SOM, National Inpatient Sample subarachnoid hemorrhage outcomes measure. Treatment refers to patients receiving aneurysm securement via surgical clipping or endovascular coiling.The bold values represent statistically significant values.

### Association of racial segregation in regional treatment facility with disparities

Segregation between White and Black patients in treatment facilities following aSAH was greatest in the Pacific (*D* index = 0.55) and Mountain (*D* index = 0.54) divisions, and lowest in the South Atlantic (*D* index = 0.39) and West South Central (*D* = 0.46) divisions. Slight variance in the *D* index within a region were noted over the five-year study, as illustrated in Supplementary Figure S2. Inter-regional comparison showed that the difference in *D* index was statistically significant across all regions over the studied period excluding comparisons between the New England and Middle Atlantic regions (Supplementary Table S3). Since 2012, the NIS has ceased providing hospital names or identifiers, offering only deidentified hospital IDs and limited characteristics such as teaching status, bed size, and hospital type. Consequently, crucial metrics for assessing hospital quality are missing. This limitation impacts our study’s use of the *D* index, as it restricts our ability to differentiate between patients treated at lower-quality and higher-quality facilities when analyzing segregation in treatment facilities.

**Figure 2:**
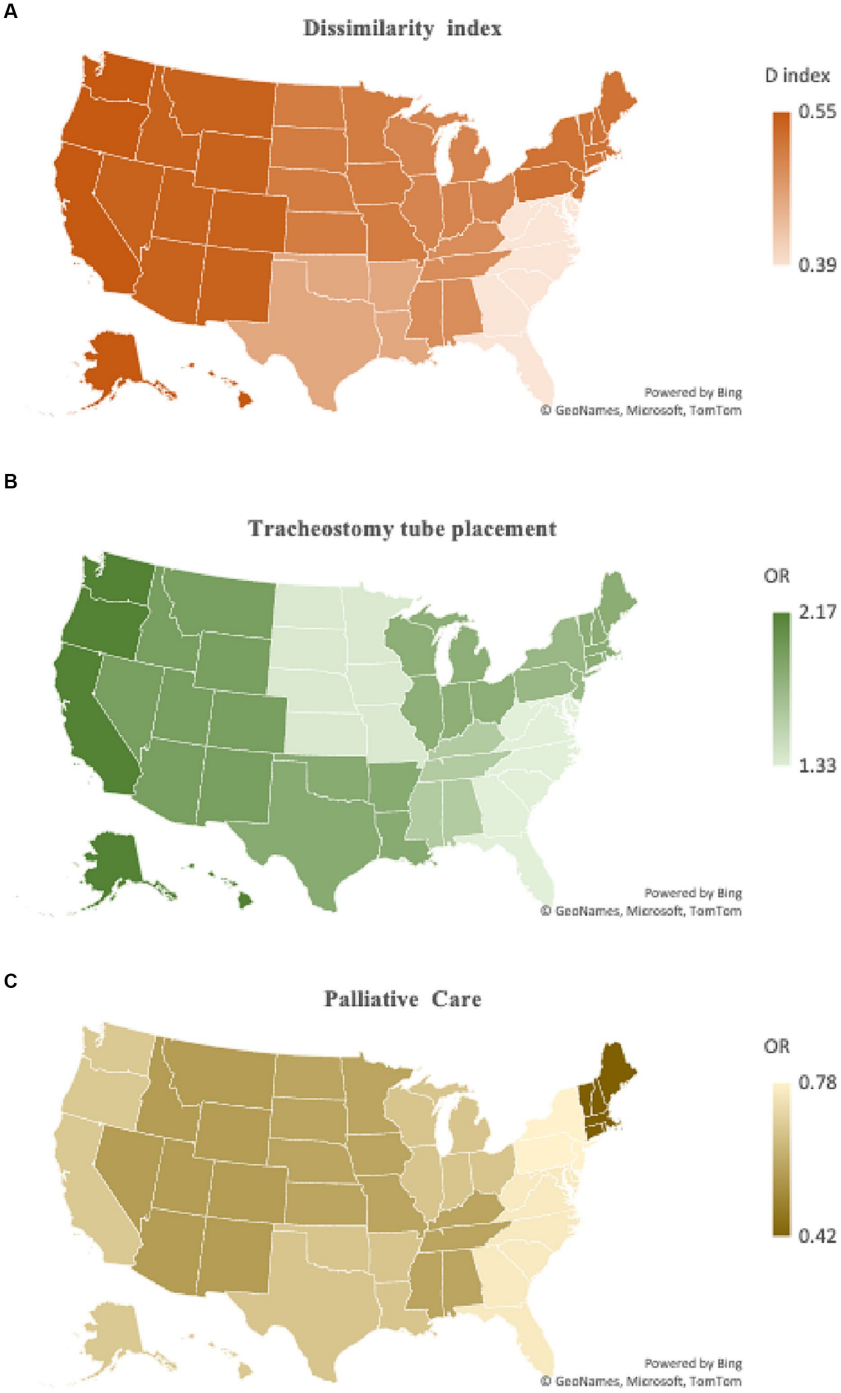
Geographical variation in dissimilarity index and association with likelihood of tracheostomy tube placement and receipt of palliative care. **(A)** A heatmap of dissimilarity index by U.S. Census region is juxtaposed to **(B)** a heatmap representation of likelihood of tracheostomy tube placement and **(C)** palliative care use for Black patients following aSAH. Regions with higher levels of segregation also had higher levels of tracheostomy tube placement for Black patients and lower palliative care consultation. This visually demonstrates the association of levels of segregation and disparities some aspects of end-of-life care.

Black patients in regions with higher levels of segregation had a higher likelihood of poor functional outcomes compared to White patients (OR 1.15, 95% CI 1.04–1.29, *p* = 0. 008). Conversely, no statistically significant difference was noted in likelihood of poor functional outcomes between Black and White patients in low *D* index regions (OR 1.07, 95% CI 0.97–1.19, *p =* 0.181). The likelihood of mortality by *D* index level (global *F* test *p* < 0.001) was higher in less segregated (OR 0.91, 95% CI 0.82–1.00, *p* = 0.044) compared to more segregated (OR 0.75, 95% CI 0.68–0.83, *p* < 0. 001) regions (*p =* 0.049). Notable differences were also seen when comparing low and high *D* index regions (global *F* test *p* < 0.001) for tracheostomy tube placement (OR 1.45, 95% CI 1.22–1.73, *p <* 0.001 vs. OR 1.87, 95% CI 1.59–2.21, *p* < 0.001) (*p* < 0. 001) and receipt of palliative care (OR 0.88, 95% CI 0.76–0.93, *p <* 0.001 vs. OR 0.67, 95% CI 0.59–0.77, *p* < 0.001) (*p* = 0.029). No statistically significant difference was found when comparing disparities between low and high *D* index region (global *F* test *p* < 0.001) for gastrostomy tube placement (OR 1.62, 95% CI 1.12–2.36, *p* = 0.011 vs. OR 1.68, 95% CI 1.46–1.93, *p* < 0.001) (*p =* 0.783), blood transfusions (OR 1.44, 95% CI 1.20–1.74, *p* < 0.001 vs. OR 1.49, 95% CI 1.24–1.79, *p* < 0.001) (*p* = 0.738), and DNR status (OR 0.68, 95% CI 0.61–0.77, *p* < 0.001 vs. OR 0.67, 95% CI 0.59–0.75, *p* < 0.001) (*p* = 0.841). The relationship between the *D* index and likelihood of tracheostomy tube placement and receiving palliative care is represented as a heatmap in Figure 2. Table 3 provides a summary of all odds ratio calculations by high versus low *D* index region.

**Table 3:** Odds Ratios for treatment, functional outcomes, morality, and end-of-life care by high versus low *D* index Region.

	**Treatment**			**NIS-SOM**			**Mortality**		
	OR	95% CI	*p*-value	OR	95% CI	*p*-value	OR	95% CI	*p*-value
High *D* index Region	1.02	0.92–1.14	0.689	1.15	1.04–1.29	**0.008**	0.75	0.68–0.83	**<0.001**
Low *D* index Region	1.14	1.03–1.26	**0.010**	1.07	0.97–1.19	0.181	0.91	0.82–1.00	**0.044**

	**Mechanical ventilation**			**Tracheostomy**			**Gastrostomy**		
	OR	95% CI	*p*-value	OR	95% CI	*p*-value	OR	95% CI	*p-*value
High *D* index Region	1.10	0.65–1.85	0.725	1.87	1.59–2.21	**<0.001**	1.68	1.46–1.93	**<0.001**
Low *D* index Region	1.44	0.85–2.46	0.180	1.45	1.22–1.73	**<0.001**	1.62	1.12–2.36	**0.011**

	**Palliative care**			**Do not resuscitate**			**Transfusions**		
	OR	95% CI	*p*-value	OR	95% CI	*p*-value	OR	95% CI	*p*-value
High *D* index Region	0.67	0.59–0.77	**<0.001**	0.66	0.59–0.75	**<0.001**	1.49	1.24–1.79	**<0.001**
Low *D* index Region	0.88	0.76–0.93	**<0.001**	0.68	0.61–0.77	**<0.001**	1.44	1.20–1.74	**<0.001**

The bold values represent statistically significant values.

## Discussion

We observed Black patients nationally underwent treatment at a slightly higher rate—and despite worse functional outcomes, Black patients were less likely to have inpatient mortality than White patients following aSAH. While the higher rate of treatment and lower mortality would at first seem to represent a positive finding, it must be taken in context. Black patients were more likely on the national level to have tracheostomy tube placement, gastrostomy tube placement, and receive blood transfusion. Black patients were less likely to have palliative care involvement or code status changed to DNR. These findings reflect a disparity in end-of-life care in that Black patients have a lower mortality and higher treatment rate because they are undergoing more life-sustaining care and less palliation in situations where the outcomes are worse. Cruz-Flores et al. report similar findings in a NIS study on intra-cerebral hemorrhages (ICH) that demonstrated Black patients with ICH are more likely to utilize lifesaving (surgical intervention), life prolonging (mechanical ventilation, tracheostomy tube, gastrostomy tube, and blood transfusions) interventions, and less likely to receive palliative and hospice care compared to White patients. These findings are posited to reflect a lower likelihood of mortality for Black patients with ICH (32).

Several explanatory factors have been cited for this global disparity within healthcare in aggressive end-of-life care (33–35). One factor is implicit bias, which refers to unconscious attitudes and stereotypes that can influence medical decision-making. Research has shown that healthcare professionals, including doctors, may hold implicit biases that contribute to perceiving Black patients as having less sensitive nerve endings and thicker skin, or feeling less empathy toward their pain, leading to the belief that they can tolerate more aggressive treatments (35, 36). Additionally, historical mistrust stemming from past instances of medical mistreatment and experimentation on Black communities can create a fear of being under-treated, prompting both patients and healthcare providers to opt for more aggressive interventions (37–39). Furthermore, disparities in access to quality healthcare and socioeconomic factors, such as limited health insurance coverage and fewer healthcare resources in predominantly Black communities, can contribute to delayed or inadequate care requiring more aggressive interventions for more severe conditions at the time of clinical presentation (40–43).

Cultural and religious considerations are pivotal in healthcare decisions, especially at the end of life. A study examining advanced cancer patients revealed that those whose spiritual needs were met by their healthcare team were more inclined to utilize hospice services and less likely to seek aggressive treatments, underscoring the significance of spirituality in these decisions (44). Interestingly, this finding was not race-specific. In contrast, a study focusing specifically on race found that while religiosity influenced the use of DNR orders among White cancer patients, it did not hold the same sway for Black patients (45). Additionally, no study to date demonstrates that Black patients rely more heavily on religious guidance than White patients when making end-of-life choices. This points to a crucial insight: while spiritual beliefs are indeed a factor, they do not singularly drive the decision-making process within the Black community, which is marked by a rich diversity of beliefs and practices shaped by personal, familial, and regional distinctions. Overstating the role of culture and religion may risk simplifying the complex interplay of influences on healthcare outcomes and divert attention from systemic barriers such as structural racism and healthcare inequities. Therefore, it’s essential to view cultural and religious beliefs as part of a wider array of determinants that collectively influence healthcare outcomes, rather than as isolated or predominant factors.

While our study found that disparities are present throughout every geographic location in the country, some regions had much higher disparities. Black patients in the Northeast (New England and Middle Atlantic) and West (Mountain and Pacific) tend to have greater disparities in life-sustaining interventions, use of palliative care, and DNR status. The South Atlantic division had the least disparities. Various factors may contribute to the observed regional variances. In this study, we demonstrated the association between disparities and segregation in treatment facilities by elucidating a relationship with disparities in treatment and clinical outcome. Segregation between Black and White patients with aSAH was greatest in regions with the largest disparities, i.e., Northeast and West. When comparing high and low segregation, more segregated regions were associated with worse functional outcomes and a lower likelihood of mortality, but notably higher likelihood of tracheostomy tube placement and decreased utilization of palliative care services.

The persistence of segregation for Black patients in treatment facilities is an amalgam of a complex interplay of historical, social, and systemic factors. Deep-rooted racial biases, discriminatory practices, and structural inequalities have perpetuated the unequal distribution of resources and opportunities within the healthcare system (46–48). Historical patterns of racial segregation, such as redlining and discriminatory housing practices, have led to the concentration of Black populations in marginalized neighborhoods with limited access to quality healthcare facilities (46–48). This spatial segregation, combined with socioeconomic disparities and inadequate healthcare infrastructure, has created barriers to equal treatment and access to care for Black individuals (49–52). Additionally, implicit biases and stereotypes among healthcare providers may contribute to differential treatment and perpetuate disparities in the delivery of healthcare services (53). Furthermore, Black patients seeking care are less likely to be transferred to a different treatment facility compared to White patients, including patients being treated for aSAH (54, 55).

To bridge the healthcare gap and combat systemic barriers, a comprehensive strategy is vital for ensuring equitable access to treatment for Black individuals, thus reducing disparities. This begins with healthcare systems conducting in-depth evaluations of facility placement and service availability, especially in highly segregated areas. Such assessments should aim to uncover and rectify care deficiencies, potentially through the strategic establishment of new facilities or the enhancement of services in existing ones within underserved Black communities.

Furthermore, forging partnerships with community organizations can improve healthcare system navigation for Black patients. Continuous bias training for healthcare providers is imperative, fostering an environment of ongoing education to combat unconscious biases that may influence patient care. In addition to this, it is crucial for healthcare professionals to embrace shared decision-making, honoring the cultural and individual preferences of Black patients, with a keen focus on end-of-life care choices. Policy reform should also focus on decentralizing high-quality care, ensuring a fair distribution of medical resources and enhancing patient transfer protocols, so that every patient, regardless of race, has access to the best possible care. Finally, culturally sensitive public health initiatives are necessary. These should provide education and resources that resonate with the Black community’s varied values and beliefs, particularly around end-of-life care. Public health campaigns can also play a role in raising awareness about the benefits of advance care planning within these communities. By implementing these steps, we can begin dismantling the deep-rooted barriers contributing to healthcare disparities, paving the way for a future where equitable care is not an ideal, but a reality for all patients.

### Limitations

Our study, while contributing important findings, is subject to several notable limitations that should be considered. A primary limitation stems from the nature of the NIS data source. Since 2012, the NIS no longer provides specific hospital names or identifiers, limiting our ability to assess the impact of individual hospital characteristics on treatment outcomes. This absence of detailed identifiers could potentially affect the external validity of our findings, as it restricts our capacity to generalize results to specific types of hospitals or geographical locations.

Furthermore, while the NIS offers a vast array of data, it primarily consists of administrative records. This reliance on administrative data can lead to a lack of nuanced clinical details and may introduce inaccuracies due to coding errors. Additionally, the large sample sizes typical of NIS data, though beneficial for statistical power, do not inherently imply clinical significance. In cases where statistical differences do not reflect clinically meaningful distinctions, the practical applicability of our findings may be limited. Hence, interpretations of our results should be made with an understanding that both statistical and clinical significance are crucial for drawing comprehensive and applicable conclusions.

## Conclusion

Nationally and regionally, our study found that Black patients undergoing aSAH treatment are more frequently subjected to life-sustaining interventions such as tracheostomy, gastrostomy, and blood transfusions, yet they receive less palliative care and fewer code status changes to DNR. This occurs despite them experiencing poorer functional outcomes, highlighting a significant disparity in treatment approaches and end-of-life care decisions Notably, the Northeast and West regions exhibited the most significant treatment disparities, correlating with the highest levels of racial segregation in healthcare facilities, while the South Atlantic division showed the least. This pattern suggests that areas with higher segregation see more pronounced disparities in both treatment and clinical outcomes.

### Previous presentation/publication

The abstract was presented at the Congress of Neurological Surgeons (CNS) annual meeting in September 2023 and awarded the CNS foundation diversity, equity, and inclusion abstract award for 2023. No parts of this study have otherwise been presented or published elsewhere.

## Data availability statement

The original contributions presented in the study are included in the article/Supplementary materials, further inquiries can be directed to the corresponding author/s.

## Author contributions

J-LK: Writing – review & editing, Writing – original draft, Visualization, Validation, Methodology, Investigation, Formal analysis, Data curation, Conceptualization. LF: Visualization, Software, Resources, Investigation, Formal analysis, Writing – review & editing, Validation, Methodology, Data curation. AB: Writing – original draft, Visualization. FD: Data curation, Writing – review & editing, Visualization, Validation. MB: Writing – review & editing, Validation, Writing – original draft, Visualization. AH: Writing – review & editing, Visualization, Validation. AR: Software, Methodology, Formal analysis, Conceptualization, Writing – review & editing, Visualization, Validation, Data curation. JP: Supervision, Writing – review & editing, Visualization, Validation. KE: Writing – original draft, Conceptualization, Writing – review & editing, Validation, Supervision. PC: Writing – original draft, Supervision. SE: Writing – review & editing, Validation, Project administration, Methodology, Data curation, Conceptualization, Supervision.
