# Supplementary material for: Association of geographical disparities and segregation in regional treatment facilities for Black patients with aneurysmal subarachnoid hemorrhage in the United States
Source: Front Public Health. 2024 May 10;12:1341212. doi: 10.3389/fpubh.2024.1341212 (PMC11121994; doi:10.3389/fpubh.2024.1341212)
Supplement: Supplementary file 2 [file Data_Sheet_2.PDF]

Supplementary Material

Association of Geographical Disparities and Segregation in Regional Treatment Facilities for Black Patients with Aneurysmal Subarachnoid Hemorrhage in the United States

Supplementary Table 1. ICD - 10 codes for variables not translated in the NIS

Variable	ICD - 10 Code
Aneurysmal SAH	160.00 - 160.09
Hypertension	I10-I15
Diabetes Mellitus	E10.618, E10.620 - E10.628, E10.65, E10.630, E10.69, E10.9, E11.618, E11.620-E11.628, E11.638, E11.00, E11.01, E11.630, E11.649, E11.65, E11.69, E11.9
Obesity	E66.01, E66.09, E66.1 - E66.9
Congestive Heart Failure	I11.0, I50.1, I50.9, I51.7
Alcohol Abuse	F10.10
Hyperlipemia	E78.0, E78.2, E78.4, E78.5
Smoking	Z87.891, F17.200
Coronary artery disease	I25.10
Arterial Fibrillation	I48.0 - I48.2, I48.91
Chronic Kidney Disease	N18.1 - N18.9
Palliatives Care	Z515
Do Not Resuscitate	Z66
Mechanical Ventilation	5A1955Z, 5A1935Z, 5A1945Z
Tracheostomy	0BH17EZ, 0BH17EZ,0BJ08ZZ
Gastrostomy	0D163J4
Blood Transfusions	30233N1

Supplementary Table 2. U.S. Census Division and State

U.S Census Division	U.S. State
New England	Maine, New Hampshire, Vermont, Massachusetts, Rhode Island, Connecticut
Middle Atlantic	New York, Pennsylvania, New Jersey
East North Central	Wisconsin, Michigan, Illinois, Indiana, Ohio
West North Central	Missouri, North Dakota, South Dakota, Nebraska, Kansas, Minnesota, Iowa
South Atlantic	Delaware, Maryland, District of Columbia*, Virginia, West Virginia, North Carolina, South Carolina, Georgia, Florida
East South Central	Kentucky, Tennessee, Mississippi, Alabama
West South Central	Oklahoma, Texas, Arkansas, Louisiana
Mountain	Idaho, Montana, Wyoming, Nevada, Utah, Colorado, Arizona, New Mexico
Pacific	Alaska, Washington, Oregon, California, Hawaii

*The District of Columbia is included in the NIS but is not a U.S. state

Supplementary Table 3. Pairwise Comparison of Dissimilarly Index by U.S. Census Division

**Census Division**		Mean Difference	*F* test *P* Value	*P* Value
**New England**	Middle Atlantic	0	**< 0.001**	1.000
	East North Central	0.02	**< 0.001**	**< 0.001**
	West North Central	0.01	**< 0.001**	**< 0.001**
	South Atlantic	0.13	**< 0.001**	**< 0.001**
	East South Central	0.03	**< 0.001**	**< 0.001**
	West South Central	0.06	**< 0.001**	**< 0.001**
	Mountain	-0.02	**< 0.001**	**< 0.001**
	Pacific	-0.03	**< 0.001**	**< 0.001**
**Middle Atlantic**	New England	0	**< 0.001**	1.000
	East North Central	0.02	**< 0.001**	**< 0.001**
	West North Central	0.01	**< 0.001**	**< 0.001**
	South Atlantic	0.13	**< 0.001**	**< 0.001**
	East South Central	0.03	**< 0.001**	**< 0.001**
	West South Central	0.06	**< 0.001**	**< 0.001**
	Mountain	-0.02	**< 0.001**	**< 0.001**
	Pacific	-0.03	**< 0.001**	**< 0.001**
**East North Central**	New England	-0.02	**< 0.001**	**< 0.001**
	Middle Atlantic	-0.02	**< 0.001**	**< 0.001**
	West North Central	-0.01	**< 0.001**	**< 0.001**
	South Atlantic	0.11	**< 0.001**	**< 0.001**
	East South Central	0.01	**< 0.001**	**< 0.001**
	West South Central	0.04	**< 0.001**	**< 0.001**
	Mountain	-0.04	**< 0.001**	**< 0.001**
	Pacific	-0.05	**< 0.001**	**< 0.001**
**East North Central**	New England	-0.01	**< 0.001**	**< 0.001**
	Middle Atlantic	-0.01	**< 0.001**	**< 0.001**
	East North Central	0.01	**< 0.001**	**< 0.001**
	South Atlantic	0.12	**< 0.001**	**< 0.001**
	East South Central	0.02	**< 0.001**	**< 0.001**
	West South Central	0.05	**< 0.001**	**< 0.001**
	Mountain	-0.03	**< 0.001**	**< 0.001**
	Pacific	-0.04	**< 0.001**	**< 0.001**
**South Atlantic**	New England	-0.13	**< 0.001**	**< 0.001**
	Middle Atlantic	-0.13	**< 0.001**	**< 0.001**
	East North Central	-0.11	**< 0.001**	**< 0.001**
	West North Central	-0.12	**< 0.001**	**< 0.001**
	East South Central	-0.10	**< 0.001**	**< 0.001**

	West South Central	-0.07	**< 0.001**	**< 0.001**
	Mountain	-0.15	**< 0.001**	**< 0.001**
	Pacific	-0.16	**< 0.001**	**< 0.001**
**East South Central**	New England	-0.03	**< 0.001**	**< 0.001**
	Middle Atlantic	-0.03	**< 0.001**	**< 0.001**
	East North Central	-0.01	**< 0.001**	**< 0.001**
	West North Central	-0.02	**< 0.001**	**< 0.001**
	South Atlantic	0.10	**< 0.001**	**< 0.001**
	West South Central	0.03	**< 0.001**	**< 0.001**
	Mountain	-0.05	**< 0.001**	**< 0.001**
	Pacific	-0.06	**< 0.001**	**< 0.001**
**West South Central**	New England	-0.06	**< 0.001**	**< 0.001**
	Middle Atlantic	-0.06	**< 0.001**	**< 0.001**
	East North Central	-0.04	**< 0.001**	**< 0.001**
	West North Central	-0.05	**< 0.001**	**< 0.001**
	South Atlantic	0.07	**< 0.001**	**< 0.001**
	East South Central	-0.03	**< 0.001**	**< 0.001**
	Mountain	-0.08	**< 0.001**	**< 0.001**
	Pacific	-0.09	**< 0.001**	**< 0.001**
**Mountain**	New England	0.02	**< 0.001**	**< 0.001**
	Middle Atlantic	0.02	**< 0.001**	**< 0.001**
	East North Central	0.04	**< 0.001**	**< 0.001**
	West North Central	0.03	**< 0.001**	**< 0.001**
	South Atlantic	0.15	**< 0.001**	**< 0.001**
	East South Central	0.05	**< 0.001**	**< 0.001**
	West South Central	0.08	**< 0.001**	**< 0.001**
	Pacific	-0.01	**< 0.001**	**< 0.001**
**Pacific**	New England	0.03	**< 0.001**	**< 0.001**
	Middle Atlantic	0.03	**< 0.001**	**< 0.001**
	East North Central	0.05	**< 0.001**	**< 0.001**
	West North Central	0.04	**< 0.001**	**< 0.001**
	South Atlantic	0.16	**< 0.001**	**< 0.001**
	East South Central	0.06	**< 0.001**	**< 0.001**
	West South Central	0.09	**< 0.001**	**< 0.001**
	Mountain	0.01	**< 0.001**	**< 0.001**

Supplementary Table 4. Univariate Logistic Regression Models of SAH Outcomes for Race and Region

	Univariate Model					
	Race			Region		
**Outcomes**	OR	95% CI	*P* Value	OR	95% CI	*P* Value
Treatment	1.34	1.25 - 1.44	< **0.001**	1.03	1.02- 1.04	< **0.001**
NIS-SOM	0.94	0.87 - 0.99	**0.026**	0.99	.98 - 1.00	**0.003**
Mortality	0.86	0.81 - 0.93	< **0.001**	1.05	1.04 - 1.05	< **0.001**
Mechanical Ventilation	1.27	1.19 - 1.34	< **0.001**	1.05	1.05 - 1.06	< **0.001**
Tracheostomy	1.94	1.74 - 2.18	< **0.001**	1.05	1.04 - 1.05	< **0.001**
Gastrostomy	1.67	1.52 - 1.83	< **0.001**	1.40	1.00 - 1.96	0.052
Blood Transfusions	1.77	1.56 - 2.01	< **0.001**	0.99	0.98 - 1.00	0.198
Palliative Care	0.61	0.56 - 0.67	< **0.001**	1.03	1.02-1.04	< **0.001**
DNR	0.57	0.53 - 0.62	< **0.001**	1.02	1.01 - 1.03	< **0.001**

Supplementary Figure 1. Flow diagram describing selection process for patients included in the study.

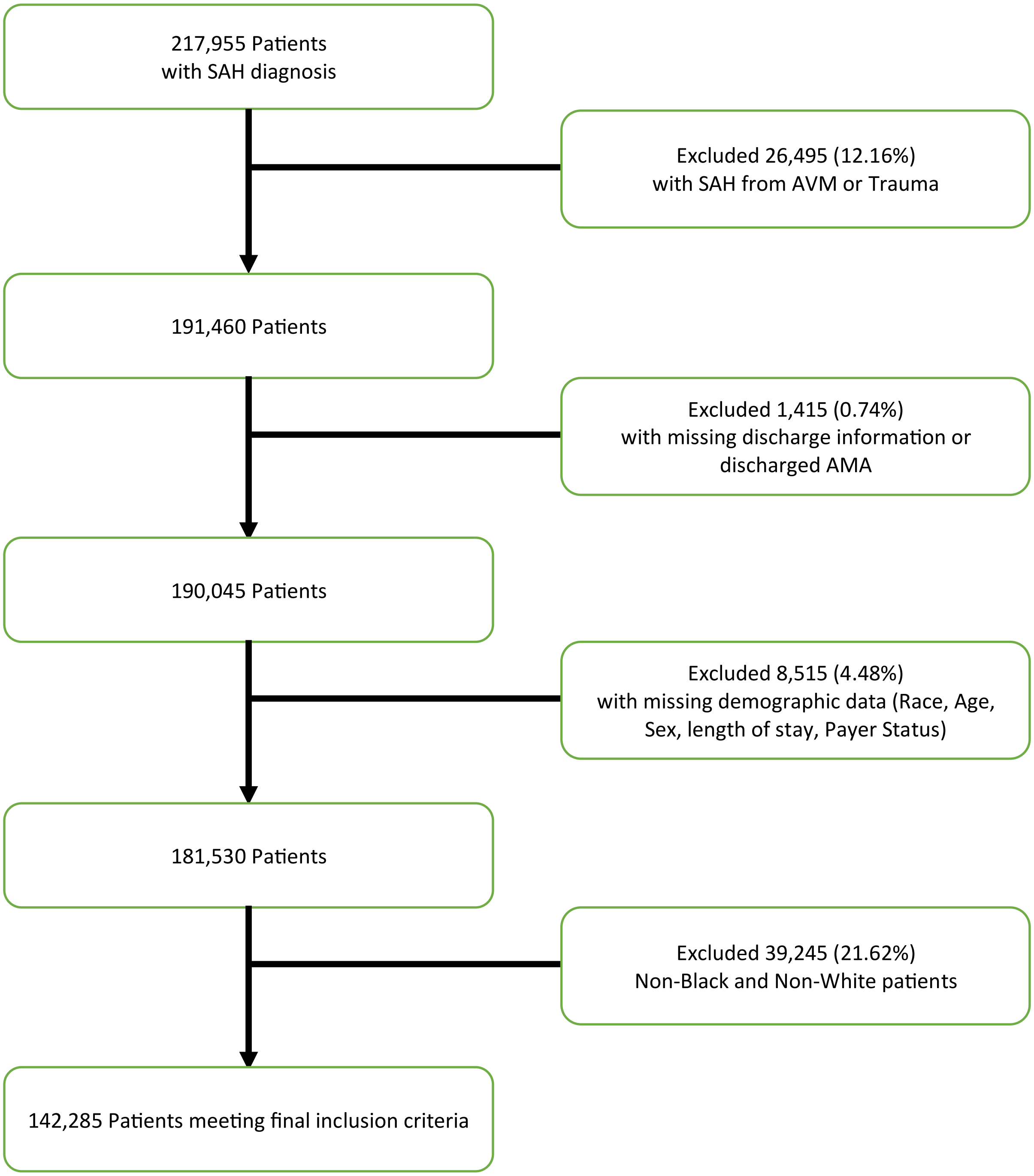

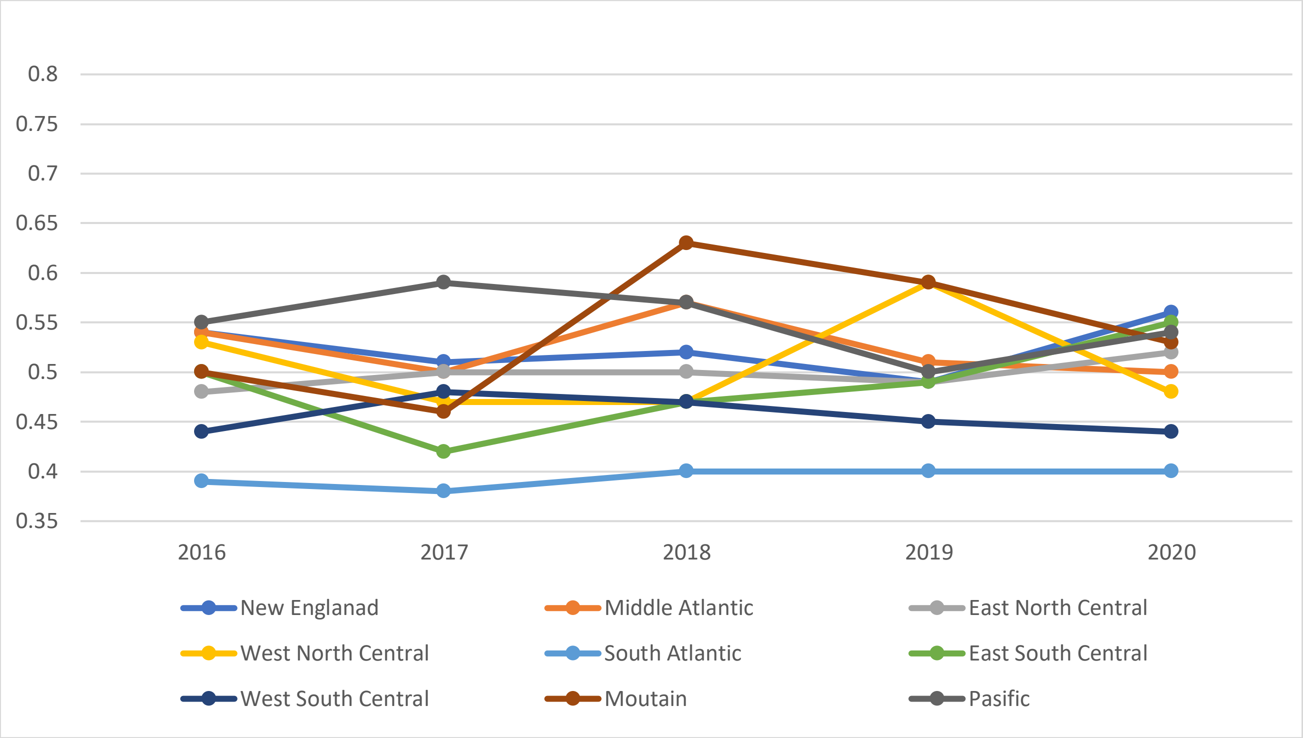

Supplementary Figure 2. Dissimilarity Index by Region 2016 - 2020. Graphical representation of dissimilarity index by U.S. Census division from 2016 – 2020. While there were yearly changes to the level of segregation in treatment facilities as measured using the dissimilarity index, the dissimilarity index within a given census division stayed roughly the same year to year. All regions demonstrated some segregation between White and Black patients in treatment facilities, however the South Atlantic region consistently had the lowest measured level of segregation, while the Mountain, Pacific, New England, and Middle Atlantic divisions had amongst the highest.
